# Run-and-pause dynamics of cytoskeletal motor proteins

**DOI:** 10.1038/srep37162

**Published:** 2016-11-16

**Authors:** Anne E. Hafner, Ludger Santen, Heiko Rieger, M. Reza Shaebani

**Affiliations:** 1 Department of Theoretical Physics, Saarland University, 66041 Saarbrücken, Germany

## Abstract

Cytoskeletal motor proteins are involved in major intracellular transport processes which are vital for maintaining appropriate cellular function. When attached to cytoskeletal filaments, the motor exhibits distinct states of motility: active motion along the filaments, and pause phase in which it remains stationary for a finite time interval. The transition probabilities between motion and pause phases are asymmetric in general, and considerably affected by changes in environmental conditions which influences the efficiency of cargo delivery to specific targets. By considering the motion of individual non-interacting molecular motors on a single filament as well as a dynamic filamentous network, we present an analytical model for the dynamics of self-propelled particles which undergo frequent pause phases. The interplay between motor processivity, structural properties of filamentous network, and transition probabilities between the two states of motility drastically changes the dynamics: multiple transitions between different types of anomalous diffusive dynamics occur and the crossover time to the asymptotic diffusive or ballistic motion varies by several orders of magnitude. We map out the phase diagrams in the space of transition probabilities, and address the role of initial conditions of motion on the resulting dynamics.

The cellular cytoskeleton is a highly dynamic and complex network of cross-linked biopolymers, which carries out essential functions in cells such as serving as tracks for motor proteins and adjusting the shape and spatial organization of the cell. The cytoskeleton enables the cell to efficiently adapt to changes in the environment. Microtubules (MTs) and actin filaments constitute the dynamic tracks for intracellular transport driven by molecular motors^1^,^2^. The cytoskeletal filaments are polar due to their structural asymmetry, with different spatial organizations. While the orientations of actin filaments are rather randomly distributed, MTs are usually growing outwards from a microtubule organizing center with their plus ends facing the cell periphery. MTs often span long distances, of the order of the cell diameter, whereas the mesh size of the actin cortex is of the order of 100 nm^3^.

Three different families of motor proteins are involved in the active intracellular transport of organelles and other cargoes: kinesins, dyneins, and myosins. Molecular motors of each family always move on a specific type of track in a particular direction^4^; while kinesins and dyneins usually move along MTs towards the plus and minus ends, respectively, different types of myosins move on actin filaments towards the plus or minus directions. Motors can bind to or unbind from the filaments. When bind to the filaments, they may perform a number of steps or remain stationary. Such binding/unbinding and moving/pausing cycles are frequently repeated during the motion of motors along the cytoskeleton. The tendency of the motor to continue its motion along the filament, called *motor processivity*, varies with the type of filament and motor^5^,^6^, and is highly sensitive to the environmental conditions such as the presence of specific binding domains or proteins^7^,^8^,^9^. Active transport on filaments makes the long-distance cargo delivery to specific targets in cells feasible, whereas the detached phases are extremely inefficient since the cytoplasm is a highly crowded environment which slows down the transfer of materials^10^,^11^. The combination of active and passive motions has been however shown to be beneficial for optimizing the first-passage properties^12^.

The motion of molecular motors involves a high degree of complexity due to the dynamics of cytoskeletal filaments, binding/unbinding and moving/pausing cycles of motion, motor-motor interactions, variations of environmental conditions, etc. The dynamics of cargoes is even more complicated, as they can be transported by teams of motors moving in different directions^13^. The movement of motor proteins can be studied at different length and time scales^11^. There have been theoretical studies focusing on the mechanistic details of stepping and the chemomechanical energy transduction process^14^,^15^,^16^. However, when considering longer length and timescales, the microscopic details of performing single steps are often ignored in the majority of the analytical studies. Instead, the dynamics of molecular motors have been modeled at the mesoscopic level, via random walk models^17^,^18^,^19^ or by solving a set of partial differential equations with appropriate boundary conditions to consider the transitions between different states of motility^20^,^21^,^22^,^23^. Stochastic two-state models of motion, consisting of altering phases of active and passive dynamics, have been widely employed to describe the motion of cytoskeletal motor proteins^24^,^25^ and swimming bacteria^26^,^27^,^28^,^29^, and locomotive patterns in other biological systems^10^,^11^,^30^,^31^. The transition probabilities between the two states are generally asymmetric and influence the efficiency of cargo delivery^28^. Interestingly, bacteria are capable of adjusting the balance between their running and tumbling states in response to the changes in environmental conditions. For example, it has been recently observed that viscoelasticity of the medium suppresses the tumbling phase and enhances the swimming speed of *E. coli*^32^.

Here, we adopt a coarse-grained approach to study the dynamics of individual non-interacting molecular motors on cytoskeleton, which enables us to identify the impact of motor processivity, structural properties of the filamentous network, and switching frequencies between the two phases of motion on the transport properties of motors. To isolate the influence of switching frequencies, we first consider the motion along a single filament and present a random walk model with two states of motility: active motion along the filament and pausing periods. The pause state emerges when the motor remains attached but stationary on the filament, or if it detaches but stays immobile at the detachment point until it attaches again. The latter case can be practically observed in laterally confined geometries, such as transport along a parallel bundle of microtubules in axons^33^,^34^,^35^. It can also happen when the detached motor carries a big vesicle or a protein complex. In this case, the diffusion constant in the cytoplasm may be even a hundred times smaller than for a single motor^36^,^37^, thus, the big complex remains practically immobile at the detachment point. Note that we do not consider here the unbinding events which are followed by passive diffusion in the cytoplasm. The formalism presented in this study can be straightforwardly extended to those run-and-tumble motions. We focus on the single (non-interacting) particle dynamics in this study. Investigation of the motion of a group of interacting motors is beyond the scope of the present study. The dynamics is more complicated in such cases as, for example, persistent motion in the presence of volume-excluded interactions between the particles leads to jammed regions or even jamming transition with increasing the particle density^29^,^38^,^39^,^40^. The effects of transition probabilities and volume exclusion on the density and traffic of motors along the filament was recently studied^24^. We assume spontaneous transitions between the two states of motility, i.e. the model is Markovian with constant probabilities. Thus, no acceleration or deceleration takes place at a switching event and the transition between the two states of motility happens instantly. Moreover, the transition probabilities are supposed to be independent. In general, the probability *κ*_*w*_ of switching from motion to pause depends on many factors, such as the applied load on the motor, cytoplasmic crowding, and the presence of microtubule-associated proteins (MAPs)^41^,^42^,^43^,^44^,^45^. Some of these factors may also affect the probability *κ*_*m*_ of switching from pause to motion, however, there are examples (such as the applied load) which do not influence *κ*_*m*_. Hence, we study the most general case, where the transition probabilities *κ*_*w*_ and *κ*_*m*_ are independent of each other. Assuming constant transition probabilities in our model results in exponential distributions for the residence times in each state, which is in agreement with the experimental findings for the distribution of active lifetimes of micron-sized beads moving along cytoskeleton^46^. We derive exact analytical expressions for the temporal evolution of the moments of displacement and show that depending on the transition probabilities between the two states, the motor can experience crossovers between different anomalous diffusive dynamics on different time scales.

In the second part of this work we study the motion of individual motor proteins on dynamic cytoskeletal filaments to disentangle the combined effects of moving/pausing transition probabilities, processivity, and structural properties of the underlying network on transport dynamics. We introduce a coarse-grained perspective to the problem and consider the motion of motors at the level of traveling between network junctions rather than individual steps along filaments. Since the distribution of directional change contains rich information about the particle dynamics^47^,^48^,^49^,^50^, we characterize the structure of the filamentous network by probability distributions *R(ϕ*) for the angle *ϕ* between intersecting filaments, and 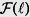 for the segment length 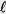 between neighboring intersections. The model consists of two states of motility: active motion and waiting at the junctions. Such waiting periods at junctions have been experimentally observed for transport along cytoskeletal networks^6^,^51^,^52^. For example, tracking the motion of lysosomes on MT networks has revealed that the particles experience even long pauses at the nodes of the network before that they can either pass through it or switch to the intersecting filament^52^. In the motion state, the motor either moves processively along the previous filament or switches to a new one. The cytoskeleton is a dynamic network due to the underlying growth and shrinkage of filaments. The dynamics even varies depending on the cell type and region^53^. Thus, the transport takes place on a continuously changing structure, which justifies the relevance of our stochastic approach as the network structure is implicitly given via the probability distributions. Within the proposed analytical framework, we prove the existence of different regimes of anomalous motion and that the motor may experience several crossovers between these regimes, as observed in various experiments of motion on cytoskeletal filaments^54^,^55^,^56^,^57^,^58^. It is also shown that the crossover times between different regimes and the asymptotic diffusion coefficient can vary widely depending on the key parameters of the model: processivity, network structure, and transition probabilities. We address the role of initial conditions of motion on the resulting dynamics^59^, and validate the theoretical predictions by performing extensive Monte Carlo simulations. By studying the evolution of the transport properties one can identify, for example, the length- and timescales on which the system can maintain a beneficial type of diffusive dynamics before a transition to another type occurs. This can be, e.g., a crossover from sub- or superdiffusion to another anomalous diffusive transport. Subdiffusion maintains concentration gradients, thus, it is beneficial for a variety of cellular functions which depend on the localization of the involved reactants^60^,^61^,^62^,^63^. On the contrary, long-distance intracellular transport needs to be even faster than the normal diffusion, since an efficient delivery of materials to their correct location within a cell is crucial for maintaining normal cellular function. This is indeed achieved by motor-driven transport along cytoskeletal filaments. Our powerful formalism enables us to study the influence of the key factors on the formation of different types of crossovers and their corresponding length- and timescales. Thus, one would be able to predict how far a desired type of transport remains efficient when the influential parameters vary because of the changes in the environment, diseases, etc. The proposed analytical approach and the results are also applicable to run-and-tumble motions in other biological as well as nonliving systems.

## Results

### Motion along a single filament

**Figure 1:**
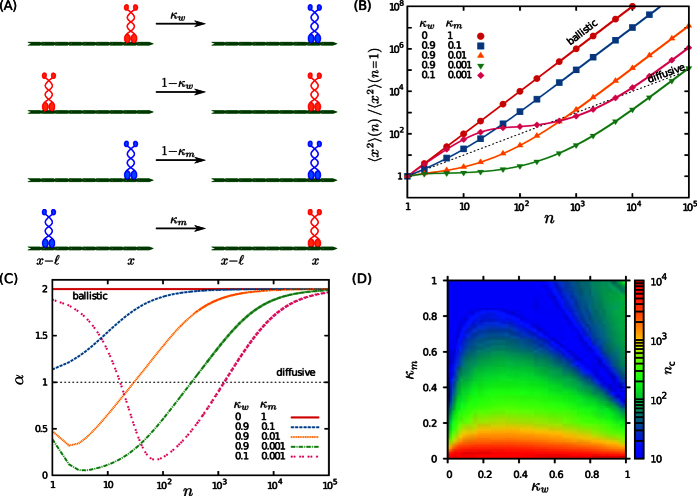
Motion on a single filament. (**A**) A schematic of the transition probabilities between pause (blue) and motion (red) states of a motor protein along a single cytoskeletal filament, as described by the set of master equations (1). The four possibilities for the motility states of two successive steps are shown separately. (**B**) MSD as a function of the step number *n* for *λ* = 1, 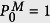, and several values of *κ*_*m*_ and *κ*_*w*_. The solid lines are obtained from the analytic expression (S17) and the symbols represent Monte Carlo simulation results. (**C**) Temporal evolution of the anomalous exponent *α* via Eq. (5), for the same parameters as in panel (**B**). (**D**) The crossover time *n*_*c*_ to the asymptotic ballistic motion for *λ* = 1 and 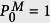 in the (*κ*_*w*_, *κ*_*m*_) phase space.

We first consider the motion of a molecular motor on a single filament in a crowded environment. For spontaneously switching motors, the stochastic motion can be described by two states of motility: (i) *ballistic motion* along the filament, and (ii) *pause* phase, where the motor remains stationary along the filament for a while (or unbinds from the filament but stays immobile until binds again). When the motor restarts the active motion, it continues towards its previous direction (unidirectional motion). The switching from motion to pause phase and vice versa happens with probabilities *κ*_*w*_ and *κ*_*m*_, respectively. The case of considerable displacements during the detached periods is not considered in the present study. The transition probabilities between the two states of motion are not necessarily symmetric in general, thus, we consider asymmetric constant transition probabilities [see Fig. 1(A)]. The assumption of constant transition probabilities leads to exponential probability distributions *P*_waiting_(*t*) and *P*_motion_(*t*) for the residence times in the waiting and motion states, respectively. For example, one can write the master equation *P*_waiting_(*t*) = *P*_waiting_(*t* − 1)(1 − *κ*_*m*_) in the waiting state, and solve it recursively to obtain 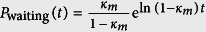. The decay exponent of the exponential distribution is proportional to ln(1 − *κ*_*m*_) and ln(1 − *κ*_*w*_) for the waiting and motion states, respectively. Thus, a smaller transition probability from state I to II, leads to a slower exponential decay for the residence time distribution in state I, resulting in a longer average lifetime in this state. At each time step, the particle either waits or performs a step of length 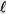 taken from a probability distribution 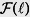. We introduce the probability densities 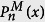 and 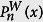 to find the walker at position *x* at time step *n* in the motion and waiting states, respectively. The temporal evolution of the process can be described by the following set of coupled master equations


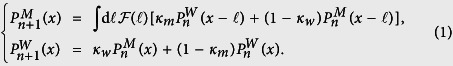


We develop a Fourier-z-transform approach^64^,^65^,^66^, which enables us to obtain exact analytical expressions for the arbitrary moments of displacement. The details of the theoretical approach can be found in the Suppl Info, where as an example, the lengthy expression for the mean squared displacement (MSD) is obtained. It can be seen from Eq. (S17) that, in addition to the transition probabilities, the results also depend on the initial conditions of motion. In derivation of Eq. (S17), denoting the probability of initially starting in the motion state by 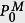, the following initial conditions are imposed


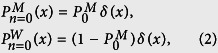


which results in 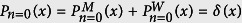. The analytical results for the time evolution of MSD are shown in Fig. 1(B) for different values of *κ_m_*, and *κ*_*w*_ parameters. The analytical predictions are also validated by performing extensive Monte Carlo simulations. The quantities of interest are obtained at discrete times by ensemble averaging over 10^6^ realizations. More details on the simulation method can be found in the Suppl Info. Several crossovers on different time scales can be observed, even though the asymptotic behavior is ballistic in all cases as expected from the unidirectional motion of the motor along the filament. In order to determine the crossovers more quantitatively, we fit the time dependence of the MSD to a power-law 〈*x*^2^〉 ~ *t*^*α*^, with *α* being the anomalous exponent. For instance, the following expression can be deduced for the initial anomalous exponent by fitting the first two steps of motion


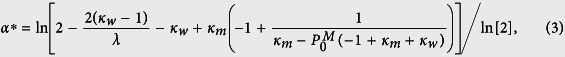


with 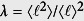 being the variance of the step-length distribution. Assuming a constant step length for simplicity (i.e. *λ* = 1), if the walker remains in the motion phase forever (i.e. *κ*_*w*_ = 0 and *κ*_*m*_ = 1) one gets α* = 2 as expected for a ballistic motion. When starting from the motion state, one has 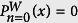 and 
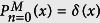
, and the initial slope follows


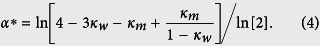


By applying the fit to successive time steps *n* and *n* + 1, the anomalous exponent as a function of time can be obtained as


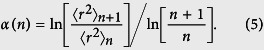


Figure 1(C) shows the time evolution of the exponent for a motor starting from the motion state. The slope *α* of the MSD curve is influenced by: (i) the initial conditions of motion 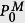 and 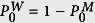, (ii) the transition probabilities *κ*_*m*_ and *κ*_*w*_ which determine the stationary probabilities 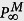 and 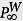 of being at each state (see Eq. (15) of the discussion section), and (iii) the asymptotic dynamics. If the initial probabilities 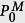 and 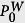 are different from the stationary ones, a transition to the stationary probabilities 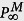 and 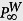 occurs, thus, *α* undergoes a crossover at short times. Another crossover happens at longer times towards the asymptotic dynamics. In the examples shown in Fig. 1(C), the initial conditions are chosen to be 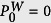 and 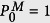, i.e. the walk always starts in the motion state. Let us consider the case of *κ*_*w*_ = 0.1 and *κ*_*m*_ = 0.001, as an example. The convergence to the stationary probabilities happens after less than 100 steps for this set of parameters (see the discussion section for details). Therefore, the first change in the curvature of *α* vs *n* in Fig. 1(C) at short times is due to the crossover from initial to stationary conditions. Starting from motion with *κ*_*w*_ = 0.1, it takes 10 steps, on average, that a transition to the waiting state happens. That is why the initial slope is superdiffusive (1 < *α*). Then, because of the very low transition probability *κ*_*m*_ = 0.001, the motor typically stays in the waiting state for long times which results in a subdiffusive dynamics (*α* < 1). Finally, the second change of the curvature of *α* at longer times (*n* ~ 1000) evidences the crossover to the asymptotic ballistic motion (*α* = 2) induced by the unidirectionality of the motion.

In the limit of n → ∞ the terms proportional to *n*^2^ dominate and the motility becomes purely ballistic. The asymptotic MSD is given by


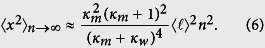


The prefactor depends on the switching probabilities. If the walker never switches to the waiting state, one recovers the relation 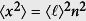 which is the fastest possible propagation. The crossover to the long-term ballistic regime is approached asymptotically. We use the distance from the exponent of ballistic motion, i.e. *δα* = |*α(n*) − 2|, and estimate the crossover time as the time step *n*_*c*_ at which *δα* drops below a threshold value *ε*. Here we report the results for *ε* = 10^−2^, however, we checked that the choice of *ε* does not affect our conclusions. As it can be seen from Fig. 1(D), *n*_*c*_ varies by several orders of magnitude in the (*κ*_*w*_, *κ*_*m*_) plane. It is expected that *n*_*c*_ increases with decreasing *κ*_*m*_, as the chance of switching to the motion state is reduced. The increase of *n*_*c*_ at large values of both probabilities *κ*_*m*_ and *κ*_*w*_ is due to frequent state oscillations which postpone the transition to asymptotic ballistic regime to longer times. We note that the length scale over which the motor reaches the asymptotic ballistic motion along a single filament is usually smaller or comparable to the cell size. Assuming that a motor protein moves with steps of size ~8 nm and a typical velocity *v* ~ 1 μm/s^67^, its displacement (until the transition to the asymptotic dynamics happens) ranges from 0.1 to 100 μm, which is comparable to typical cell sizes. Thus, the motor can practically experience the transition to long-term ballistic motion along a single microtubule within the cell body for a considerable range of the transition probabilities *κ*_*w*_ and *κ*_*m*_.

### Motion on a dynamic filamentous network

The theoretical framework can be generalized to describe active motion of particles on filamentous networks. We adopt a coarse-grained approach in which the motion of motor proteins is modeled as a persistent random walk on the intersections of cytoskeletal filaments. The structure of the network is characterized by the probability distributions *R(ϕ*) for the angle *ϕ* between intersecting filaments, and 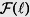 for the segment length 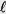 between neighboring intersections. Furthermore, a parameter *p* is introduced to take the processivity of molecular motors into account. The particle either waits at each time step (waiting state) or walks with a step length 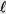 (motion state). In the latter state, the motor either continues along the previous filament with probability *p* or chooses a new filament with probability 1−*p*. Similar to motion on a single filament, the transition probabilities *κ*_*m*_ and *κ*_*w*_ between the two states are assumed to be asymmetric and constant. The parameter *κ*_*m*_ (*κ*_*w*_) denotes the switching probability from waiting to motion (motion to waiting) state. The probability density functions 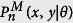 and 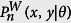 denote the probability to find the walker at position (*x*, *y*) along the direction *θ* at time step *n* in the motion and waiting states, respectively. Here a 2D system is considered for simplicity. For extension of the approach to 3D see ref. 48. The evolution of the process is described by the following set of coupled master equations


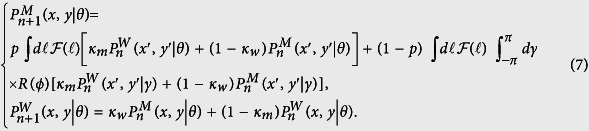


**Figure 2:**
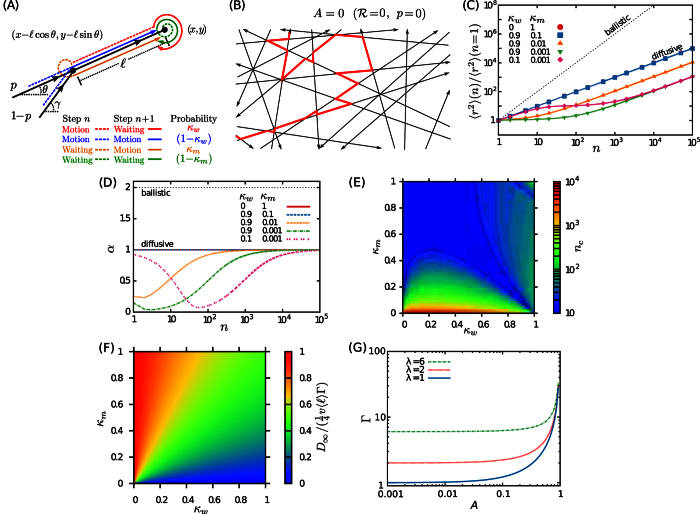
Non-processive motion on random actin networks. (**A**) Schematic of the path on a cytoskeletal network during two consecutive steps of motion, as described by the coupled set of master equations (7). (**B**) A typical sample trajectory (red lines) of a non-processive walker (*p* = 0) on a random filamentous structure (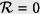). (**C**) MSD as a function of the step number *n* for *λ* = 1, 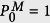, *A* = 0, and several values of *κ*_*m*_ and *κ*_*w*_. The solid lines correspond to the analytical expression (S45) and the symbols represent the simulation results. (**D**) Temporal evolution of the anomalous exponent *α* via Eq. (5), for the same parameters as in panel (**C**). (**E,F**) Phase diagrams of (**E**) the crossover time *n*_*c*_ to the asymptotic diffusive regime, and (**F**) the long-term diffusion constant *D*_∞_, scaled by 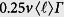, via Eq. (12) for *A* = 0, *λ* = 1, and 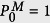 in the (*κ*_*w*_, *κ*_*m*_) plane. (**G**) The scale parameter 
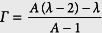
 vs *A* for several values of *λ*.

**Figure 3:**
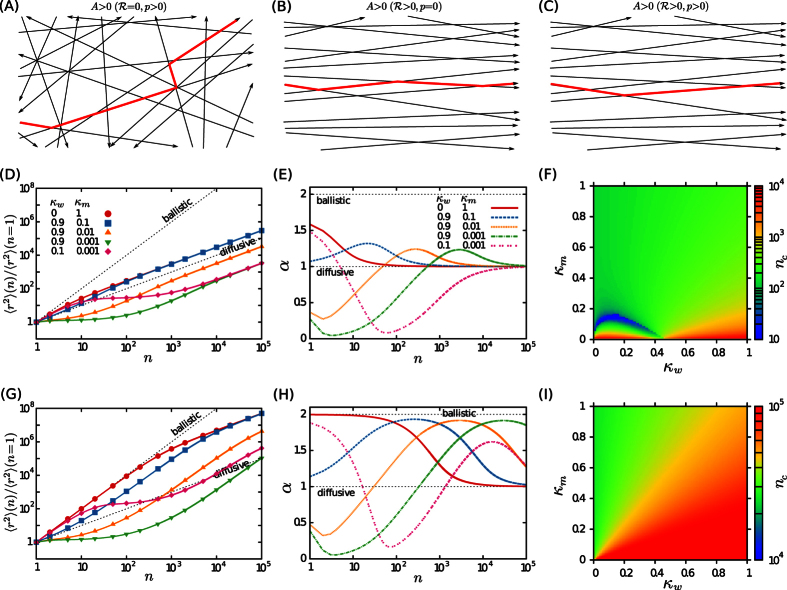
Motion on microtubule networks/Processive motion. In the upper panels, typical sample trajectories (red lines) are shown for positive values of the parameter *A*: (**A**) a processive motion (*p* > 0) on a random filamentous structure (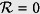), (**B**) a non-processive motion (*p* = 0) on a bundle of relatively parallel filaments (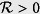), and (**C**) a processive motion on a bundle of relatively parallel filaments (which corresponds to an extremely high value of *A*). In the middle and lower panels, the results for *A* = 0.5 and 0.99 are shown, respectively. The latter value is relevant for a processive motion on highly aligned filaments, while the former case corresponds to either a non-processive motion on moderately aligned filaments or, alternatively, active motion with moderate processivity on isotropic networks. (**D,G**) MSD in terms of the step number *n* for *λ* = 1, 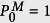, and several values of *κ*_*m*_ and *κ*_*w*_. The solid lines (symbols) correspond to analytical results via Eq. (S45) (simulation results). (**E,H**) Time evolution of the anomalous exponent *α* for the same parameter values as in panels (**D,G**). (**F,I**) Crossover time *n*_*c*_ to the asymptotic diffusive dynamics in the case of *λ* = 1 and 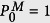 in the (*κ*_*w*_, *κ*_*m*_) phase space.

Assuming isotropic initial conditions


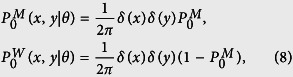


which leads to 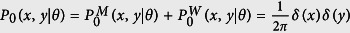, one can follow the proposed Fourier-z-transform formalism and calculate arbitrary moments of displacement. The exact analytical expression for the MSD, which is the main quantity of interest, is given in Eq. (S45). The interplay between transition probabilities, motor processivity, initial conditions of motion, and structure of the underlying network lead to a variety of anomalous transport dynamics on different time scales. The structure of the network is characterized by the relative variance of 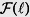 (i.e. 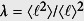) and the Fourier transform 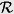 of *R(ϕ*), which quantify the heterogeneity and anisotropy of the environment, respectively. 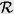 ranges from −1 to 1, with 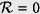 for a completely random structure, and positive (negative) values for an increased probability for motion in the near forward (backward) directions. The processivity *p* and anisotropy 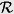 parameters always appear combined as 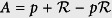 in the solution; thus, by varying *A* one can separately study the effects of *p* or 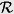 on the transport of motors. We first choose *A* = 0 in Fig. 2, corresponding to a non-processive motion (*p* = 0) on an isotropic structure such as actin filament networks (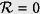), and then study positive values *A* = 0.5 and *A* = 0.99 in Fig. 3. These latter choices can e.g. correspond to either a non-processive motion (*p* = 0) on aligned filaments (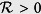) such as radially organized MT networks, or an active motion (*p* > 0) on an isotropic actin network (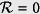).

As the phase space of the system is entangled, we restrict ourselves to initially starting from the motion state (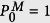) in this section, and elaborate on the role of initial conditions in the discussion section. A wide range of different types of anomalous motion can be observed on varying the transition probabilities *κ*_*w*_ and *κ*_*m*_. The possible crossovers at short and intermediate time scales are even more diverse than those observed for the motion on a single filament. A similar approach as in the previous section is followed to obtain the time evolution of the anomalous exponent and identify the crossovers. The long term dynamics is diffusive in all cases as the directional memory is short ranged and the walker eventually gets randomized on the network. However, we note that the asymptotic diffusive motion might be observable only on time and length scales which are not accessible in experiments. By fitting to the power-law 〈*x*^2^〉 ~ *t*^*α*^, the initial anomalous exponent is obtained as


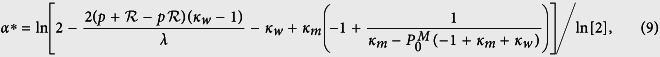


which in the case of *κ*_*w*_ = 0 and *κ*_*m*_ = 1 reduces to


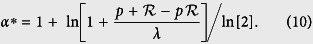


For example, one gets α* = 1 for non-processive motion on random actin networks in the absence of waiting phases, which is expected to be diffusive on all time scales. From Eq. (9) one can also see the impact of the initial conditions of motion and heterogeneity of the network on the exponent.

In the long-time limit, the terms linear in *n* dominate, thus, the motility becomes purely diffusive. From Eq. (S45), the MSD in the limit of *n* → ∞ is given by


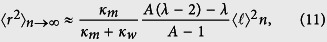


from which the asymptotic diffusion constant can be determined as


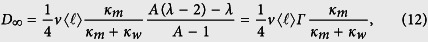


with 
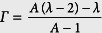
 and *v* being the average motor velocity. Figure 2(F) shows how *D*_∞_ varies in the space of transition probabilities. While decreasing *κ*_*w*_ or increasing *κ*_*m*_ enhances the diffusion coefficient, changing the probabilities in the opposite direction leads to a strong localization. Taking the dependence of *Γ* on the heterogeneity parameter *λ* into account [see Fig. 2(G)], it can be seen that *D*_∞_ varies by several orders of magnitude by varying the key parameters of the problem. Interestingly, the long-term diffusion constant does not depend on the initial conditions.

To estimate the transition time *n*_*c*_ to asymptotic diffusive regime, we follow the convergence of the anomalous exponent towards 1, and determine *n*_*c*_ as the earliest time step at which the exponent difference drops below a threshold *ε*, i.e.


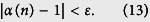


The results shown in Figs 2(E), 3(F) and 3(I), reported for *ε* = 10^−2^, reveal that *n*_*c*_ varies over several orders of magnitude. On an actin filament network with an average mesh size 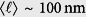 and a typical motor velocity *v* ~ 1 μm/s, *n*_*c*_ ranges from 0.1 seconds to more than 100 seconds, which might be beyond the possible time window of experiments, thus, all different regimes of motion are not necessarily realized in practice. It is difficult to make a direct comparison with the reported experiments due to the lack of the required data. Still, let us consider the motion of a motor protein with steps of size 8 nm and a typical velocity v ~ 1 μm/s along microtubules, as an example. Starting from the motion state, if we adjust the transition probabilities to *κ*_*w*_ = 0.9 and *κ*_*m*_ = 0.01 and the processivity to *A* = 0.99, our formalism predicts a crossover at short times (~100 ms) from sub- (with 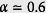) to superdiffusion (with 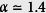), which is quantitatively comparable to the transition reported in experiments on the bidirectional organelle transport along microtubules^54^. To compare with other analytical and numerical studies, we first adjust our model parameters to those of a single-state (i.e. *κ*_*w*_ = 0 and *κ*_*m*_ = 1) active motion along a square lattice (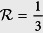) and compare the initial anomalous exponents with those obtained from a random velocity model (RVM) for active transport on a similar structure^68^. We get initial slopes ranging between 1.41 and 2 depending on the processivity of the walker, which are in agreement with the initial slopes shown in Fig. (6) of the RVM paper (ranging within [4/3, 2] upon varying the processivity). However, there are major differences between the two models: (i) The disorder is quenched in the RVMs, in contrast to the dynamic random networks in our model, and (ii) the combination of the stochastic processes in the RVMs results in very different anomalous transport on intermediate and long timescales and the asymptotic dynamics is not a normal diffusion necessarily. As a final comparison, an anomalous exponent α* = 1.44 was recently reported in simulations where the particle experiences altering phases of active motion along random filaments and passive diffusion in the cytoplasm^69^. The displacements of the particle are considerably large in the latter phase, of the order of those in the active run phase. We can approximate this motion with a single-state non-processive motion (*κ*_*w*_ = 0, *κ*_*m*_ = 1, *p* = 0) in our model, where the turning-angle distribution of the particle is a combination of a uniform distribution (for the passive diffusion phase) and a delta function in the forward direction (for the run phase along filaments). This leads to a quantitatively comparable effective initial exponent 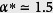.

**Figure 4:**
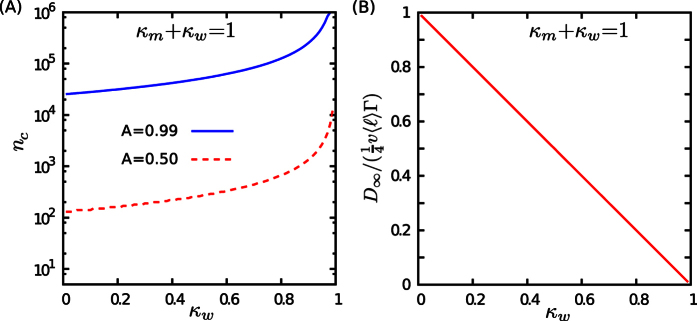
(**A**) The crossover time *n*_*c*_ to the asymptotic diffusive dynamics for different values of parameter *A*, and (**B**) the asymptotic diffusion constant *D*_∞_ in terms of *κ*_*w*_, when the transition probabilities are constrained to *κ*_*m*_ + *κ*_*w*_ = 1.

We note that the assumption of independent transition probabilities *κ*_*w*_ and *κ*_*m*_ enables us to study the most general case and obtain the quantities of interest in the (*κ*_*m*_, *κ*_*w*_) phase space. Indeed, this phase diagram contains the results for any functionality between *κ*_*w*_ and *κ*_*m*_. In some cases, the influential factors affect the transition probabilities in opposite directions. For example, increasing the density of MAPs on one hand enhances the steric inhibition of the motor motility (thus increases *κ*_*w*_) and on the other hand decreases the chance of restarting the motion (i.e. decreases *κ*_*m*_). In the absence of quantitative experimental studies, here we consider the choice *κ*_*m*_ + *κ*_*w*_ = 1 as a particular case of inversely related transition probabilities; the increase of one of them is accompanied by the decrease of the other one. The particular consequence of choosing *κ*_*m*_ + *κ*_*w*_ = 1 is that the system immediately undergoes the crossover to the equilibrated state probabilities 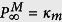 and 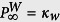, as can be seen from Eq. (14) in the next section. Therefore, the initial anomalous exponent α* does not depend on the choice of the initial state probabilities 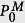 and 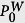: From Eq. (9), one obtains 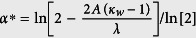. Moreover, the asymptotic diffusion constant via Eq. (12) can be expressed in terms of just one of the transition probabilities, e.g. 
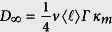
. Figure 4 shows how *D*_∞_ and the crossover time to the asymptotic diffusion vary in terms of the transition probabilities when they are constrained to *κ*_*m*_ + *κ*_*w*_ = 1. The line *κ*_*m*_ + *κ*_*w*_ = 1 divides the phase diagram into two subdomains: In the region below (above) this line, one or both of *κ*_*w*_ and *κ*_*m*_ can be rather small (large). As we explained in the previous section, smaller (larger) transition probabilities lead to slower (faster) exponential decays for the residence time distributions, resulting in longer (shorter) average lifetimes for each state of motility.

## Discussion

**Figure 5:**
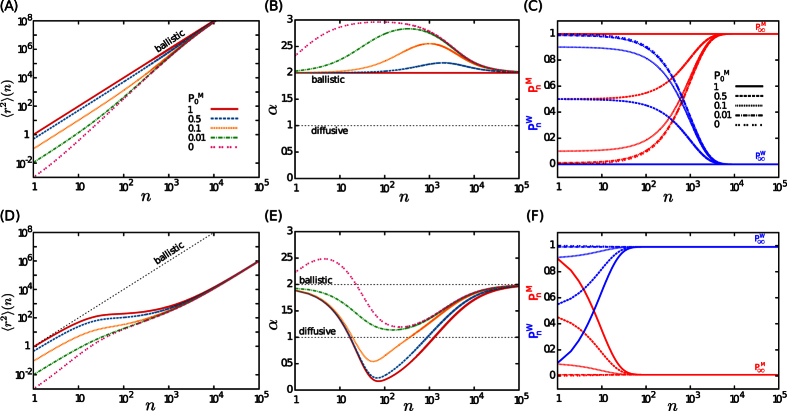
Influence of initial conditions. The dynamics on a single filament in the absence of waiting (*κ*_*w*_ = 0, upper panels) and for weak switching probabilities to waiting state (*κ*_*w*_ = 0.1, lower panels) are compared. (**A,D**) MSD as a function of the step number *n* for *κ*_*m*_ = 0.001, *λ* = 1, and different probabilities 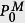 of initially starting in the motion state. (**B,E**) Temporal evolution of the anomalous exponent *α(n*) for the same set of parameters as in panels (**A,D**). (**C,F**) Evolution of the Markov chain probabilities 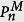 (red lines) and 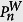 (blue lines), via Eq. (14). The stationary probabilities are given by Eq. (15).

In the previous sections we showed that the transport dynamics of molecular motors depend on the initial conditions of motion. This can be more clearly seen in Fig. 5(A,D), where the MSD for the motion along a single filament is plotted for a given set of *κ*_*w*_ and *κ*_*m*_ parameters and for different probabilities 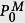 of initially starting in the motion state (see also Fig. 5(B,E) for the influence of initial conditions on the anomalous exponent *α*). Indeed, the probabilities of finding the walker in each of the two states of motility at time *n*, i.e. 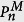 and 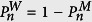, are controlled by the transition probabilities *κ*_*w*_ and *κ*_*m*_ at long times. Thus, the influence of initial conditions gradually weakens as 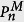 and 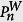 converge towards their stationary values. The sequence of motion and waiting states can be considered as a discrete time Markov chain with transition probabilities *κ*_*m*_ and *κ*_*w*_. Denoting the initial probabilities by 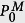 and 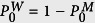, it can be verified that the time evolution of these probabilities follows


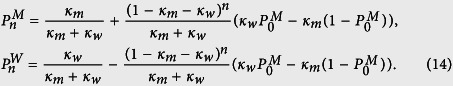


The probabilities eventually converge to the stationary values


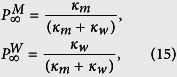


thus, if the process is initially started with these probabilities, the system is already equilibrated. Otherwise, the dynamics at short times is influenced by the choice of initial conditions of motion until the system relaxes towards stationary state, as shown in Fig. 5(C,F).

**Figure 6:**
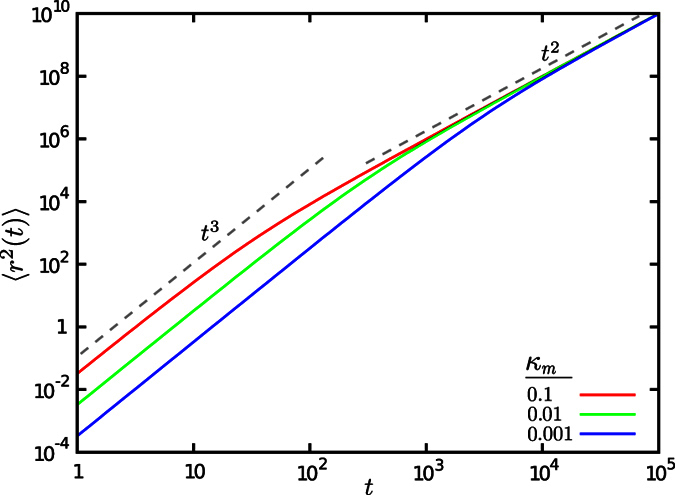
MSD for the motion on a single filament, starting from the initial condition 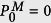 and without any switching from the motion to waiting state, i.e. *κ*_*w*_ = 0. The results are shown for *λ* = 1 and several values of *κ*_*m*_.

By varying the probability 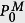 of initially starting in the motion state, we find regimes with an anomalous exponent greater than 2 in Fig. 5(B,E). To understand the origin of this peculiar behavior we note that the presented MSD is indeed an ensemble averaged quantity. When starting from the initial probability 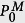 which is smaller (larger) than the equilibrated value 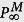, an acceleration (deceleration) due to the injection of more (less) particles from the waiting to the motion state occurs. This is especially more obvious for the case of motion along a single filament with 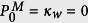. In this case, all particles are in the waiting state initially, and no transition to the waiting state happens during the process, thus, all particles switch to the motion state gradually according to the transition probability *κ*_*m*_. By injection of more particles into the ballistic motion state, the exponents greater than 2 appear, as shown in Fig. 6 (see also the Supplementary Figure S2). The exponent converges to 2 as the system approaches the stationary state. Figure 6 shows that the initial slope of the ensemble averaged MSD is nearly 3 for this set of the parameter values, which can be explained as follows. Because of the constant transition probabilities, the residence time in the waiting state is exponentially distributed. The particles of the ensemble, which are all in the waiting phase initially, switch to motion at exponentially distributed times *t*_0_, i.e. 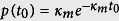. As soon as a particle switches the state, it performs a ballistic motion with velocity *v*, thus


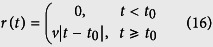


and we obtain the ensemble averaged MSD by integration over all possible transition times until time *t*


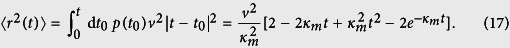


By Taylor expansion around *t* = 0, one finds 
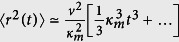
, which verifies that the initial anomalous exponent is nearly 3. A similar procedure can be applied to obtain the initial exponents for other sets of parameter values, as those shown in Fig. 5(B,E).

In summary, we developed a general analytical framework to study the dynamics of molecular motors on cytoskeleton, which enabled us to identify the influence of filament network structure, moving/pausing probabilities, and motor processivity on the transport properties of motors. The flexibility of our coarse-grained formalism allowed us to consider different filamentous structures, from a single filament to a complex network of biopolymers characterized by its structural heterogeneity and anisotropy. We obtained exact analytical expressions for the arbitrary moments of displacement, and verified that the motors display a wide range of different types of motion due to the interplay between motor processivity, structural properties of filamentous network, and transition probabilities between the two states of motility. One observes that multiple crossovers occur between different types of anomalous transport at short and intermediate timescales, and that the crossover time to the asymptotic diffusive or ballistic motion varies by several orders of magnitude. We also addressed how the initial conditions of motion affect the resulting dynamics.

In the analysis presented in this work, the transitions between the two states of motion were assumed to be spontaneous, thus, the motion of motors was described by a Markovian process. However, switching between the states might be not necessarily spontaneous, and the process can be non-Markovian in general, e.g. if the residence time in a state affects its switching probability to the other state. These generalizations can be handled within our proposed analytical framework enabling to deal with different types of environments. The analytical formalism is also applicable to study similar dynamics in other biological as well as nonliving systems, such as the run-and-tumble motion of bacteria in biological media. It is worthwhile to mention that the motility in the passive state may not be negligible in general. The dynamics in such a case can be described by altering states of motion with different velocities and run times.

## Methods

We develop an analytical Fourier-z-transform formalism to describe the persistent motion of motor proteins along cytoskeletal filaments with stochastic pausing periods. A random walk in discrete time and continuous space with two states of motility is introduced. The walker either performs a persistent motion on filaments or waits when faces obstacles or tumbles in the crowded cytoplasm. By introducing the probability density functions 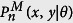 and 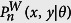 for the probability to find the walker at position (*x*, *y*) along the direction *θ* at time step *n* in the motion or waiting states, the temporal evolution of the process can be described by a set of coupled master equations, where the transitions from motion to waiting state and vice versa (denoted by *κ*_*w*_ and *κ*_*m*_, respectively) are assumed to be asymmetric and constant. Then, arbitrary moments of displacement can be calculated by applying Fourier and z transforms on the set of master equations. Finally, the quantities of interest can be obtained in the real space and time by means of inverse transforms. A detailed description of the analytical approach can be found in Suppl Info.

## Additional Information

**How to cite this article**: Hafner, A. E. *et al.* Run-and-pause dynamics of cytoskeletal motor proteins. *Sci. Rep.* **6**, 37162; doi: 10.1038/srep37162 (2016).

**Publisher’s note**: Springer Nature remains neutral with regard to jurisdictional claims in published maps and institutional affiliations.

## Supplementary Material

Supplementary Information
