# Supplementary material for: Run-and-pause dynamics of cytoskeletal motor proteins
Source: Sci Rep. 2016 Nov 16;6:37162. doi: 10.1038/srep37162 (PMC5111058; doi:10.1038/srep37162)
Supplement: Supplementary Information [file srep37162-s1.pdf]

Supplementary Information to Run-and-pause dynamics of cytoskeletal motor proteins

Anne E. Hafner[1], Ludger Santen[1], Heiko Rieger[1], M. Reza Shaebani[1]

[1] *Department of Theoretical Physics, Saarland University, 66041 Saarbrücken, Germany*

We develop an analytical framework in which the stochastic motion of cytoskeletal motor proteins along filaments is considered as a random walk in discrete time and continuous space with two states of motility: (i) *(anti-)persistent motion* on filaments and (ii) *waiting* when tumbling in the crowded cytoplasm.

Motion along a single filament

First we study the active motion of a motor protein on a single one-dimensional cytoskeletal filament. The movement is frequently interrupted by detachment events, and the resulting excursion times in the crowded cytoplasm are considered as waiting periods along the filament. When the motor attaches again to the filament, it continues its unidirectional motion until the next detachment event happens. The transition probabilities between the two states of motion are not necessarily symmetric in general, thus, we consider asymmetric constant transition probabilities κ_m and κ_w from waiting to motion state and vice versa, respectively. At each time step, the particle either waits or performs a step of length ℓ taken from a probability distribution $\mathscr{F}(\ell)$. We introduce the probability densities $P_n^M(x)$ and $P_n^W(x)$ to find the walker at position x at time step n in the motion and waiting state, respectively. The temporal evolution of the process can be described by the following set of coupled master equations

$$\begin{cases} P_{n+1}^M(x) = \int \mathrm{d}\ell\, \mathscr{F}(\ell)\big[\kappa_m P_n^W(x{-}\ell) + (1-\kappa_w) P_n^M(x{-}\ell)\big], \\ P_{n+1}^W(x) = \kappa_w P_n^M(x) + (1{-}\kappa_m) P_n^W(x). \end{cases} \tag{1}$$

In the following, we briefly demonstrate a Fourier-z-transform approach[38,39,40] to obtain arbitrary moments of displacement. The Fourier transform of $P_n^j(x)$ is defined as

$$P_n^j(\omega) \equiv \int \mathrm{d}x\; e^{i\omega x} P_n^j(x), \tag{2}$$

from which one can calculate the moments of displacement

$$\langle x^k \rangle_n^j \equiv \int \mathrm{d}x\; x^k P_n^j(x) = (-i)^k \frac{\partial^k P_n^j(\omega)}{\partial \omega^k}\bigg|_{\omega=0}. \tag{3}$$

For example, the first two moments are given by

$$\langle x \rangle_n^j = -i \frac{\partial P_n^j(\omega)}{\partial \omega}\bigg|_{\omega=0}, \tag{4}$$

$$\langle x^2 \rangle_n^j = (-i)^2 \frac{\partial^2 P_n^j(\omega)}{\partial \omega^2}\bigg|_{\omega=0}. \tag{5}$$

By Fourier transforming Eqs. (1), we obtain the set of master equations in Fourier space

$$P_{n+1}^M(\omega) = \int \mathrm{d}\ell\, \mathscr{F}(\ell)\, e^{i\omega\ell}\, \big[\kappa_m P_n^W(\omega) + (1-\kappa_w) P_n^M(\omega)\big], \tag{6}$$

$$P_{n+1}^W(\omega) = \kappa_w P_n^M(\omega) + (1-\kappa_m) P_n^W(\omega). \tag{7}$$

Then, using the z-transform technique, these coupled linear equations can be solved. The z-transform of a function T_n^j is defined as

$$T^j(z) = \sum_{n=0}^{\infty} T_n^j z^{-n}, \tag{8}$$

which results in

$$P^M(z,\omega) = \frac{z-(1-\kappa_m)}{\kappa_w} P^W(z,\omega) - \frac{z}{\kappa_w} P_{n=0}^W(\omega), \tag{9}$$

$$P^W(z,\omega) = \frac{\frac{z}{\langle e^{i\omega\ell}\rangle} - (1-\kappa_w)}{\kappa_m} P^M(z,\omega) - \frac{z}{\langle e^{i\omega\ell}\rangle\, \kappa_m} P_{n=0}^M(\omega), \tag{10}$$

with $\langle e^{i\omega\ell}\rangle = \int \mathrm{d}\ell\, \mathscr{F}(\ell)\, e^{i\omega\ell}$. Denoting the probability of initially starting in the motion state by P_0^M, we apply the following initial conditions

$$P_{n=0}^M(\omega) = P_0^M, \tag{11}$$
$$P_{n=0}^W(\omega) = 1 - P_0^M, \tag{12}$$
$$P_{n=0}(x) = P_{n=0}^M(x) + P_{n=0}^W(x) = \delta(x), \tag{13}$$

and solve the coupled set of Eqs. (9) and (10) to obtain

$$P(z,\omega) = P^M(z,\omega) + P^W(z,\omega) = \frac{z\left[\langle e^{i\omega\ell}\rangle\left(P_0^M - 1\right)(\kappa_m + \kappa_w - 1) - P_0^M\,(\kappa_m + \kappa_w - 1) - z\right]}{\langle e^{i\omega\ell}\rangle\,(z - z\kappa_w + \kappa_m + \kappa_w - 1) - z\,(z + \kappa_m - 1)}. \tag{14}$$

The z-transform of Eqs. (4) and (5) leads to the following expressions for the moments of displacement in z-space

$$\langle x\rangle^j(z) = \sum_{n=0}^{\infty} z^{-n}\langle x\rangle_n^j = (-i)\left.\frac{\partial P^j(z,\omega)}{\partial\omega}\right|_{\omega=0}, \tag{15}$$
$$\langle x^2\rangle^j(z) = \sum_{n=0}^{\infty} z^{-n}\langle x^2\rangle_n^j = (-i)^2\left.\frac{\partial^2 P^j(z,\omega)}{\partial\omega^2}\right|_{\omega=0}. \tag{16}$$

Finally, by taking into account the contributions of both states of motility and inverse z-transforming the z-space moments, we derive exact analytical expressions for arbitrary moments of displacement in real time. For example, the time evolution of the MSD as a function of the transition probabilities and initial conditions reads

$$\begin{aligned}
\langle x^2\rangle_n =& 1/(\kappa_m+\kappa_w)^4 \times \\
&\Bigg[\langle \ell^2\rangle(\kappa_m+\kappa_w)^2\Big(-\kappa_m + P_0^M\kappa_m + \kappa_m^2 - P_0^M\kappa_m^2 + \kappa_m^2 n + P_0^M\kappa_w + \kappa_m\kappa_w - 2P_0^M\kappa_m\kappa_w + \kappa_m n\kappa_w - P_0^M\kappa_w^2 \\
&\qquad + (1-\kappa_m-\kappa_w)^{(1+n)}(\kappa_m - P_0^M\kappa_m - P_0^M\kappa_w)\Big) \\
&+ \langle\ell\rangle^2\Big[\kappa_m^4 n(1 - 2P_0^M + n) + 2\kappa_m^3\Big(-1 + P_0^M - n + P_0^M n - \kappa_w + P_0^M\kappa_w - 3P_0^M n\kappa_w + n^2\kappa_w \\
&\qquad + (-1+P_0^M)(1-\kappa_m-\kappa_w)^n\big(-1 + (-1+n)\kappa_w\big)\Big) \\
&\quad + 2P_0^M(-1+\kappa_w)\kappa_w^2\Big(-1 + \kappa_w + (1-\kappa_m-\kappa_w)^n\big(1 + (-1+n)\kappa_w\big)\Big) \\
&\quad + \kappa_m^2\Big(2 - 2P_0^M + 4\kappa_w + 4P_0^M n\kappa_w - 4\kappa_w^2 + 6P_0^M\kappa_w^2 - 3n\kappa_w^2 - 6P_0^M n\kappa_w^2 + n^2\kappa_w^2 + 2(1-\kappa_m-\kappa_w)^n\big(-1 + P_0^M \\
&\qquad + (-2 + n - P_0^M n)\kappa_w + (-2 + 3P_0^M)(-1+n)\kappa_w^2\big)\Big) \\
&\quad + 2\kappa_m\kappa_w\Bigg(-(-1+\kappa_w)\Big(-2 + \big(1 + P_0^M(-3+n) + n\big)\kappa_w\Big) + (1-\kappa_m-\kappa_w)^n\Big(2 \\
&\qquad + \kappa_w\big(-3 + 3P_0^M + n - 2P_0^M n + (-1+3P_0^M)(-1+n)\kappa_w\big)\Big)\Bigg)\Big]\Bigg].
\end{aligned} \tag{17}$$

Motion on a dynamic filamentous network

The theoretical framework can be generalized to describe active motion of particles on 2D filamentous networks (see also Ref. [27] for extension to 3D). Here, a coarse-grained approach is adopted in which the motion of motor proteins is modeled as a persistent random walk on the intersections of cytoskeletal filaments. The structure of the network is characterized by the probability distributions $R(\phi)$ for the angle ϕ between intersecting filaments, and $\mathscr{F}(\ell)$ for the segment length ℓ between neighboring intersections. Furthermore, a parameter p is introduced to take the processivity of molecular motors into account. The particle either waits at each time step (waiting state) or walks with a step length ℓ (motion state). In the latter state, the motor either continues along the previous filament with probability p or chooses a new filament with probability $1{-}p$. Similar to motion on single filament, the transition probabilities κ_m and κ_w between the two states are assumed to be asymmetric and constant. The parameter κ_m (κ_w) denotes the switching probability from waiting to motion (motion to waiting) state. The probability density functions $P_n^M(x,y|\theta)$ and $P_n^W(x,y|\theta)$ denote the probability to find the walker at position (x,y) along the direction θ at time step n in the motion and waiting state, respectively. The temporal evolution of the process is described by the following set of coupled master equations

$$\begin{cases}
P_{n+1}^M(x,y|\theta) = p\int d\ell\,\mathscr{F}(\ell)\Big[\kappa_m P_n^W\big(x',y'\big|\theta\big) + (1-\kappa_w)\,P_n^M\big(x',y'\big|\theta\big)\Big] \\
\qquad\qquad + (1-p)\int d\ell\,\mathscr{F}(\ell)\int_{-\pi}^{\pi} d\gamma\, R(\phi)\Big[\kappa_m P_n^W\big(x',y'\big|\gamma\big) + (1-\kappa_w)\,P_n^M\big(x',y'\big|\gamma\big)\Big], \\
P_{n+1}^W(x,y|\theta) = \kappa_w P_n^M(x,y|\theta) + (1-\kappa_m)P_n^W(x,y|\theta),
\end{cases} \tag{18}$$

where the turning angle is given by $\phi=\theta-\gamma$, and the position at time step n is denoted by $x'=x-\ell\cos\theta$ and $y'=y-\ell\sin\theta$. The terms on the right-hand side of the first equation correspond, respectively, to persistent motion along the previous filament and switching to a new filament with probabilities p and $1-p$. We follow a similar Fourier-z-transform technique as introduced in the previous section to obtain arbitrary moments of displacement. The Fourier transform of $P_n^j(x,y|\theta)$ is defined as

$$P_n^j(\boldsymbol{\omega}|m) \equiv \int_{-\pi}^{\pi} \mathrm{d}\theta\, e^{im\theta} \int \mathrm{d}y \int \mathrm{d}x\; e^{i\boldsymbol{\omega}\cdot\boldsymbol{r}} P_n^j(x,y|\theta), \tag{19}$$

and the moments of displacement can be calculated as

$$\langle x^{k_1} y^{k_2} \rangle_n^j \equiv \int \mathrm{d}\theta \int \mathrm{d}y \int \mathrm{d}x\; x^{k_1} y^{k_2} P_n^j(x,y|\theta) = (-i)^{k_1+k_2} \frac{\partial^{k_1+k_2} P_n^j(\omega_x,\omega_y|m=0)}{\partial \omega_x^{k_1} \partial \omega_y^{k_2}}\Bigg|_{(\omega_x,\omega_y)=(0,0)}. \tag{20}$$

Fourier transforming the first equation of the set of master equations (18) leads to

$$P_{n+1}^M(\omega,\alpha|m) = \sum_{k=-\infty}^{\infty} i^k\, e^{-ik\alpha}\big(p+s\mathscr{R}(m+k)\big) \int \mathrm{d}\ell\, \mathscr{F}(\ell) J_k(\omega\ell) \Big[\kappa_m P_n^W(\omega,\alpha|m+k) + (1-\kappa_{_\mathrm{w}}) P_n^M(\omega,\alpha|m+k)\Big], \tag{21}$$

and the second equation of (18) can be similarly transformed. In Eq. (21), we used the Fourier transform of the intersection-angle distribution

$$\mathscr{R}(m) = \int_{-\pi}^{\pi} \mathrm{d}\phi\; e^{im\phi} R(\phi), \tag{22}$$

and the kth order Bessel's function

$$J_k(z) = \frac{1}{2\pi i^k} \int_{-\pi}^{\pi} \mathrm{d}\theta\; e^{iz\cos\theta} e^{-ik\theta}. \tag{23}$$

In contrast to the one-dimensional case, the procedure to obtain the moments of displacement is more complicated here, and contains further steps. To this aim, one needs to expand the Fourier transform of the probability $P_n^j(\omega,\alpha|m)$ as a Taylor series

$$P_n^j(\omega,\alpha|m) = Q_{0,n}^j(\alpha|m) + i\,\omega \int \mathrm{d}\ell\, \mathscr{F}(\ell)\,\ell\; Q_{1,n}^j(\alpha|m) - \frac{1}{2}\omega^2 \int \mathrm{d}\ell\, \mathscr{F}(\ell)\,\ell^2\; Q_{2,n}^j(\alpha|m) + \dots, \tag{24}$$

and use the Taylor expansion coefficients to get the moments of displacement as

$$\langle x \rangle_n^j = \int \mathrm{d}\ell\, \mathscr{F}(\ell)\,\ell\; Q_{1,n}^j(0|0) \quad = \langle \ell \rangle\, Q_{1,n}^j(0|0), \tag{25}$$

$$\langle y \rangle_n^j = \int \mathrm{d}\ell\, \mathscr{F}(\ell)\,\ell\; Q_{1,n}^j\Big(\frac{\pi}{2}\Big|0\Big) = \langle \ell \rangle\, Q_{1,n}^j\Big(\frac{\pi}{2}\Big|0\Big), \tag{26}$$

$$\langle x^2 \rangle_n^j = \int \mathrm{d}\ell\, \mathscr{F}(\ell)\,\ell^2\; Q_{2,n}^j(0|0) \quad = \langle \ell^2 \rangle\, Q_{2,n}^j(0|0), \tag{27}$$

$$\langle y^2 \rangle_n^j = \int \mathrm{d}\ell\, \mathscr{F}(\ell)\,\ell^2\; Q_{2,n}^j\Big(\frac{\pi}{2}\Big|0\Big) = \langle \ell^2 \rangle\, Q_{2,n}^j\Big(\frac{\pi}{2}\Big|0\Big). \tag{28}$$

The following recursion relations for the Taylor expansion coefficients result from expanding both sides of master equations (18) and collecting all terms with the same power in ω

$$Q_{0,n+1}^W(\alpha|m) = \kappa_w Q_{0,n}^M(\alpha|m) + (1-\kappa_m) Q_{0,n}^W(\alpha|m), \tag{29}$$

$$Q_{0,n+1}^M(\alpha|m) = \Big[\kappa_m Q_{0,n}^W(\alpha|m) + (1-\kappa_w) Q_{0,n}^M(\alpha|m)\Big]\big(p+s\mathscr{R}(m)\big), \tag{30}$$

$$Q_{1,n+1}^W(\alpha|m) = \kappa_w Q_{1,n}^M(\alpha|m) + (1-\kappa_m) Q_{1,n}^W(\alpha|m), \tag{31}$$

$$\begin{aligned} Q_{1,n+1}^M(\alpha|m) =&\, \kappa_m \Big\{ Q_{1,n}^W(\alpha|m)\big(p+s\,\mathscr{R}(m)\big) + \frac{1}{2}\Big[e^{i\alpha} Q_{0,n}^W(\alpha|m-1)\big(p+s\,\mathscr{R}(m-1)\big) + e^{-i\alpha} Q_{0,n}^W(\alpha|m+1)\big(p+s\,\mathscr{R}(m+1)\big)\Big]\Big\} \\ &+ (1-\kappa_w) \Big\{ Q_{1,n}^M(\alpha|m)\big(p+s\,\mathscr{R}(m)\big) + \frac{1}{2}\Big[e^{i\alpha} Q_{0,n}^M(\alpha|m-1)\big(p+s\,\mathscr{R}(m-1)\big) + e^{-i\alpha} Q_{0,n}^M(\alpha|m+1)\big(p+s\,\mathscr{R}(m+1)\big)\Big]\Big\}, \end{aligned} \tag{32}$$

$$Q^W_{2,n+1}(\alpha|m) = \kappa_w Q^M_{2,n}(\alpha|m)+(1-\kappa_m)Q^W_{2,n}(\alpha|m), \tag{33}$$

$$\begin{aligned}
Q^M_{2,n+1}(\alpha|m) = \kappa_m \Big\{ \Big[\frac{1}{2}Q^W_{0,n}(\alpha|m)+Q^W_{2,n}(\alpha|m)\Big](p+s\,\mathcal{R}(m)) + \frac{\langle\ell\rangle^2}{\langle\ell^2\rangle}\Big[e^{i\alpha}Q^W_{1,n}(\alpha|m-1)(p+s\,\mathcal{R}(m-1)) \\
+e^{-i\alpha}Q^W_{1,n}(\alpha|m+1)(p+s\,\mathcal{R}(m+1))\Big]+\frac{1}{4}e^{2i\alpha}Q^W_{0,n}(\alpha|m-2)(p+s\,\mathcal{R}(m-2)) \\
+\frac{1}{4}e^{-2i\alpha}Q^W_{0,n}(\alpha|m+2)(p+s\,\mathcal{R}(m+2))\Big\} \\
+(1-\kappa_w)\Big\{ \Big[\frac{1}{2}Q^M_{0,n}(\alpha|m)+Q^M_{2,n}(\alpha|m)\Big](p+s\,\mathcal{R}(m)) + \frac{\langle\ell\rangle^2}{\langle\ell^2\rangle}\Big[e^{i\alpha}Q^M_{1,n}(\alpha|m-1)(p+s\,\mathcal{R}(m-1)) \\
+e^{-i\alpha}Q^M_{1,n}(\alpha|m+1)(p+s\,\mathcal{R}(m+1))\Big]+\frac{1}{4}e^{2i\alpha}Q^M_{0,n}(\alpha|m-2)(p+s\,\mathcal{R}(m-2)) \\
+\frac{1}{4}e^{-2i\alpha}Q^M_{0,n}(\alpha|m+2)(p+s\,\mathcal{R}(m+2))\Big\}.
\end{aligned} \tag{34}$$

The Taylor expansion coefficients in z-space are obtained by applying the z-transform to Eqs.(29)-(34). For example, for the first Taylor expansion coefficient one finds

$$Q^W_0(z,\alpha|m) = \frac{1}{G^m(z)}\Big[z\kappa_w Q^M_{0,n=0}(\alpha|m) + z\big[z-(1-\kappa_w)\big(p+s\,\mathcal{R}(m)\big)\big]Q^W_{0,n=0}(\alpha|m)\Big], \tag{35}$$

$$Q^M_0(z,\alpha|m) = \frac{1}{G^m(z)}\Big[z\kappa_{_m}\big(p+s\,\mathcal{R}(m)\big)Q^W_{0,n=0}(\alpha|m) + z\big[z-(1-\kappa_m)\big]Q^M_{0,n=0}(\alpha|m)\Big], \tag{36}$$

with

$$G^m(z) = [z-(1-\kappa_m)][z-(1-\kappa_w)(p+s\,\mathcal{R}(m))] - \kappa_m\kappa_w(p+s\,\mathcal{R}(m)). \tag{37}$$

Then the components of the moments of displacement in z-space, e.g. the x component of the first two moments, read

$$\langle x\rangle^j(z) = \sum_{n=0}^{\infty} z^{-n}\langle x\rangle^j_n = \sum_{n=0}^{\infty} z^{-n}\langle\ell\rangle\, Q^j_{1,n}(0|0) = \langle\ell\rangle\, Q^j_1(z,0|0), \tag{38}$$

$$\langle x^2\rangle^j(z) = \sum_{n=0}^{\infty} z^{-n}\langle x^2\rangle^j_n = \sum_{n=0}^{\infty} z^{-n}\langle\ell^2\rangle\, Q^j_{2,n}(0|0) = \langle\ell^2\rangle\, Q^j_2(z,0|0). \tag{39}$$

Finally, an inverse z-transform is needed to get the moments of displacement back in real time. The useful inverse z-transformations are

$$\frac{z}{z-1} \Longleftrightarrow 1, \tag{40}$$

$$\frac{z}{(z-1)^2} \Longleftrightarrow n, \tag{41}$$

$$\frac{z}{(z-1)(z-a)} \Longleftrightarrow \frac{1-a^n}{1-a}, \tag{42}$$

$$\frac{z}{(z-1)(z-a)(z-b)} \Longleftrightarrow \frac{a^n(1-b)+b^n(a-1)+b-a}{(a-b)(b-1)(1-a)}, \tag{43}$$

$$\frac{z}{(z-1)^2(z-a)} \Longleftrightarrow \frac{a^n-1+n(1-a)}{(1-a)^2}. \tag{44}$$

By taking into account the contributions of both states of motility and the initial conditions, e.g. starting from the motion state with probability P^M_0 with isotropic initial direction, our analytical approach allows for the calculation of arbitrary moments of displacement. For instance, introducing $A = p+\mathcal{R}-p\mathcal{R}$ and $\lambda = \langle\ell^2\rangle/\langle\ell\rangle^2$ one gets the following exact analytical expression of the mean squared displacement

$$
\begin{aligned}
\langle r^2\rangle_n = &-\frac{\langle \ell^2\rangle}{(A-1)^2\,\lambda}\times \\
&\Bigg[2A+2AP_0^M-2A^2P_0^M+P_0^M\lambda-2AP_0^M\lambda+A^2P_0^M\lambda+\frac{(A-1)\big(A(\lambda-2)-\lambda\big)\kappa_m}{(\kappa_m+\kappa_w)^2}-\frac{(A-1)\,P_0^M\big(A(\lambda-2)-\lambda\big)}{\kappa_m+\kappa_w} \\
&-\frac{(A-1)\big(A(\lambda-2)-\lambda\big)\kappa_m\,(1+n)}{\kappa_m+\kappa_w}+\frac{(A-1)\big(A(\lambda-2)-\lambda\big)(1-\kappa_m-\kappa_w)^{1+n}\big((P_0^M-1)\kappa_m+P_0^M\kappa_w\big)}{(\kappa_m+\kappa_w)^2} \\
&-\frac{2^{-n}A}{\sqrt{\big(\kappa_m-1+A(\kappa_w-1)\big)^2+4A(\kappa_m+\kappa_w-1)}}\times\Bigg[\left(1+A-\kappa_m-A\kappa_w+\sqrt{\big(\kappa_m-1+A(\kappa_w-1)\big)^2+4A(\kappa_m+\kappa_w-1)}\right)^n\times \\
&\left(1-2P_0^M-\kappa_m+2P_0^M\kappa_m+2P_0^M\kappa_w+\sqrt{\big(\kappa_m-1+A(\kappa_w-1)\big)^2+4A(\kappa_m+\kappa_w-1)}+A\big(2\kappa_m+\kappa_w-1-2P_0^M(\kappa_m+\kappa_w-1)\big)\right) \\
&+\left(1+A-\kappa_m-A\kappa_w-\sqrt{\big(\kappa_m-1+A(\kappa_w-1)\big)^2+4A(\kappa_m+\kappa_w-1)}\right)^n\times \\
&\left(2P_0^M-1+\kappa_m-2P_0^M\kappa_m-2P_0^M\kappa_w+\sqrt{\big(\kappa_m-1+A(\kappa_w-1)\big)^2+4A(\kappa_m+\kappa_w-1)}+A\big(1-2\kappa_m-\kappa_w+2P_0^M(\kappa_m+\kappa_w-1)\big)\right)\Bigg]\Bigg].
\end{aligned}
\tag{45}
$$

SIMULATION METHOD

In the previous section, we derived exact analytical expressions for the moments of displacement. In view of the complexity of the analytical calculations, we perform Monte Carlo simulations to verify their correctness. In this section, we explain how the MSD (as the main quantity of interest) is evaluated in our simulations. Considering a one-dimensional motion along a single filament for simplicity, the MSD after n steps can be evaluated as

$$\langle x_n^2\rangle = \int x^2\,P_n(x)\;\mathrm{d}x, \tag{46}$$

where $P_n(x)$ denotes the probability of finding the particle at position x after n steps. In Monte Carlo simulations, the MSD is practically calculated by averaging over an ensemble of particle trajectories, i.e. the integral is approximated by the average over a sequence of independent displacements $\{x_n(i)\}_{i=1,\ldots,N}$ as

$$\langle x_n^2\rangle = \lim_{N\to\infty}\frac{1}{N}\sum_{i=1}^{N}x_n(i)^2, \tag{47}$$

where the sum runs over all realizations. In our simulations, the total number of realizations N is set to 10^6. The basic scheme of the Monte Carlo algorithm is shown in supplementary figure 1. Initially, the step length distribution $\mathscr{F}(\ell)$, the transition probabilities κ_m, κ_w, the initial-state probability P_0^M are given as inputs. At each step of the simulation, a decision is made between remaining in the current state of motility or switching to the other state, according to the probabilities κ_m and κ_w. Consequently, the position of the particle is either unchanged (for being in the waiting state) or updated according to a random step length drawn from the distribution $\mathscr{F}(\ell)$ (for being in the motion state). After reaching the last time step, the next particle starts at x_0=0 and the time loop is repeated. Finally, when the realization loop ends, the mean squared displacement over all realizations is calculated.

The case of a dynamic filamentous network is more complicated, as the directional correlation needs to be considered as well. The main differences with the algorithm of motion along a single filament appear in the position update procedure (see supplementary figure 1). The current direction of motion θ_i is stored at each time step i. If the next state of motility is "waiting", the position as well as the direction remain unchanged. If the particle decides to move in the next time step, then it should be also decided whether the motion is processive or a turning happens. For a processive motion, only the position will be updated according to the distribution $\mathscr{F}(\ell)$. For the case of turning, the direction θ is also updated by adding a stochastic rotation angle ϕ drawn from the distribution $R(\phi)$. Thus, both of the position and direction of motion will be updated in this case. The loops over time steps and realizations will be repeated similarly to the case of motion along a single filament.

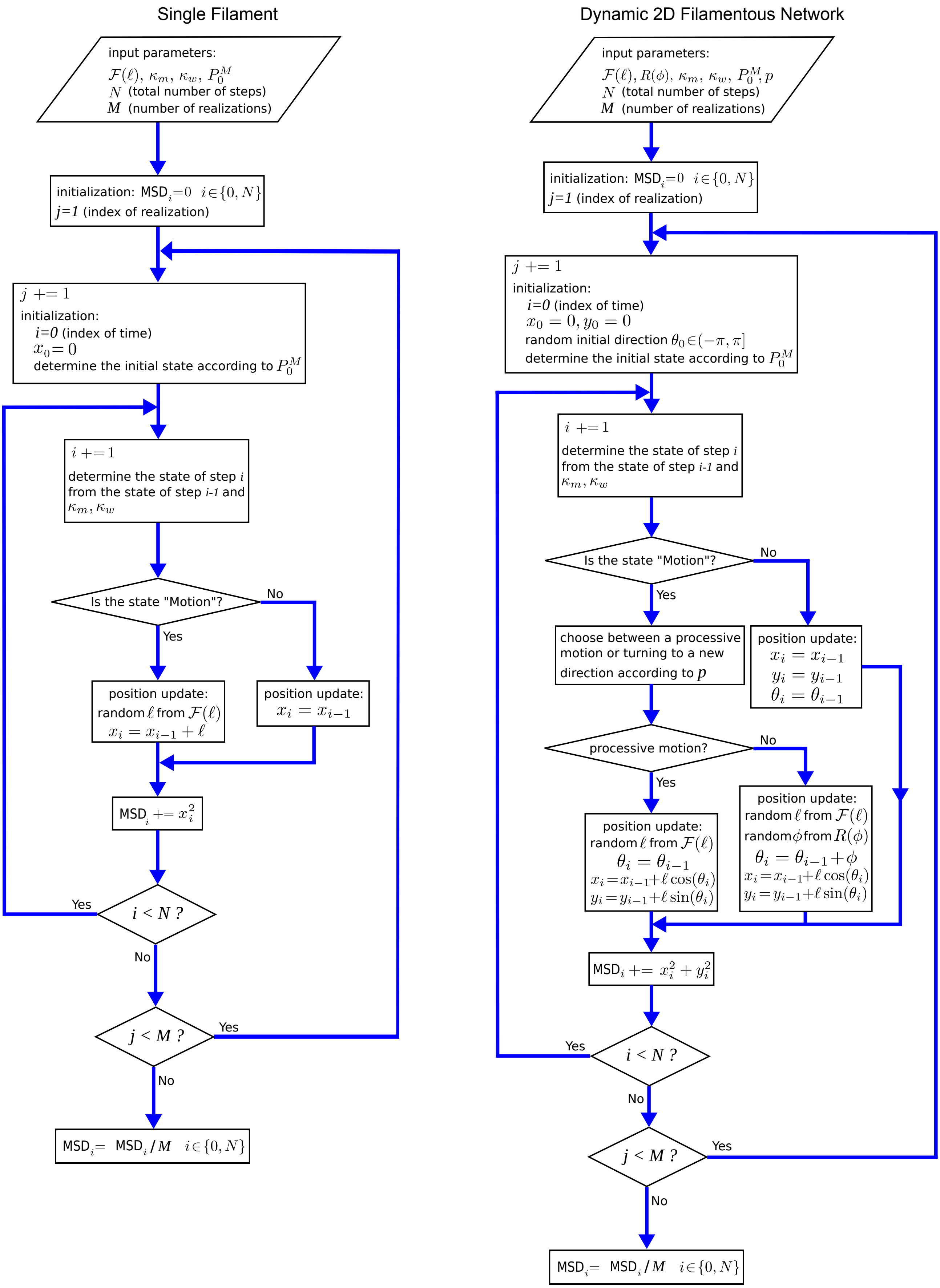

Supplementary Figure S1: Flowchart of the main steps of the simulation for motion along (left) a single filament, and (right) a 2D dynamic filamentous network.

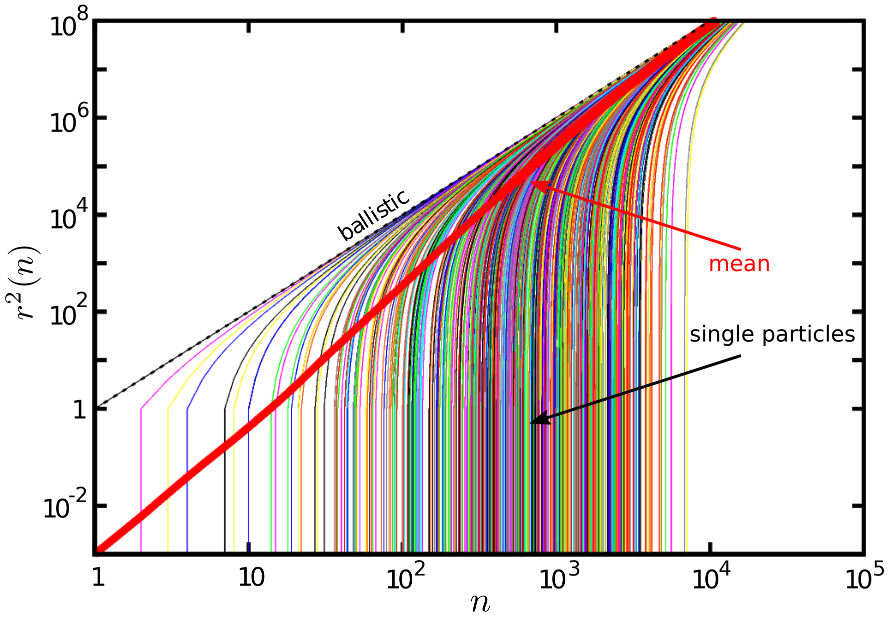

Supplementary Figure S2: MSD of individual particles switching from the waiting to motion state at different times on a single filament. The parameter values are λ=1, κ_w=0, κ_m=0.001 and P_0^M=0. The thick solid line indicates the ensemble averaged MSD, $\langle r^2 \rangle(n)$.
